# Solvent–Solvent Fractionation of Ora-Pro-Nobis (*Pereskia aculeata*) Leaves Enhances Polyphenol Enrichment and Red Blood Cell Protection against Oxidative and Osmotic Stress

**DOI:** 10.1021/acsomega.5c12684

**Published:** 2026-03-19

**Authors:** Thiago M. Cruz, Yasmin Stelle, Daniel Granato, Mariza B. Marques

**Affiliations:** † Department of Chemistry, 67883 State University of Ponta Grossa, Av. Carlos Cavalcanti 4748, 84030-900, Ponta Grossa, Brazil; ‡ School of Science, 1410 Auckland University of Technology, Auckland 1010, New Zealand

## Abstract

Ora-pro-nobis is a bioactive food plant widely distributed across Latin America and the Caribbean, with its biological activities largely attributed to leaf polyphenols. Solvent–solvent fractionation is an effective approach to enriching these compounds. Dichloromethane (FD), ethyl acetate (FAE), *n*-butanol (FB), and aqueous (FAq) fractions were obtained from the crude extract (CE), and their phenolic profiles, chemical antioxidant activity (CAA), and red blood cell (RBC) protection were evaluated. FAE contained the highest levels of total phenols (65 mg of GAE/g), flavonoids (56 mg of CE/g), chlorogenic acid (5147 μg/g), *p*-coumaric acid (10625 μg/g), ferulic acid (18482 μg/g), ellagic acid (36402 μg/g), and quercetin (1491 μg/g). In contrast, FB was the richest in rutin (3889 μg/g), and FAq was the richest in gallic acid (880 μg/g). In CAA assays, FAE exhibited superior activity in DPPH (79 mg of AAE/g), ABTS (114 mg of AAE/g), and FRAP (152 mg of AAE/g), while CE was most effective in Fe^2+^-chelation (96 mg of EDTAE/g). In TBARS assays, all samples protected RBCs comparably (61.5–68.8% inhibition), except FD (45.3%). All fractions inhibited oxidative hemolysis in a dose-dependent manner and mitigated protein oxidation. They also reduced erythrocyte osmotic fragility by lowering H_50_ (0.401–0.424%) and osmotic hemolysis (45.2–74.3%). Overall, FAE concentrated the highest load of bioactive compounds and emerged as the most promising fraction for nutraceutical development.

## Graphical abstract

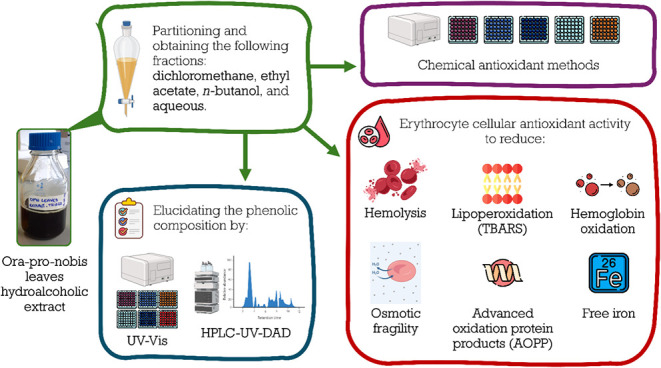

## 1 Introduction

Ora-pro-nobis (OPN) is a popular name for *Pereskia aculeata* Miller, a Cactaceae plant originally from Panama and the north of South America,^1^ but is widely distributed along Central America, South America, the Caribbean, and North America.^2^ OPN is a food plant with leaves that are used in folk medicine to treat anemia, diabetes, hypertension, cancer, osteoporosis, stomach issues, and intestinal constipation.^3,4^ Furthermore, its extracts present antioxidant,^5,6^ antinociceptive,^7^ neuroprotective, anti-inflammatory,^8^ antimicrobial,^9^ and antihemolytic^10^ activities, and bioactive substances, such as phenolic compounds, confer these biological activities to these leaves. Phenolic compounds are substances whose structure is composed of at least one aromatic ring with one or more hydroxyls as substituents.^11^ Phenolic compounds previously identified in OPN leaves include ferulic acid, quercetin, catechin, and caffeic acid.^12^

One of the most effective approaches for isolating bioactive compounds is solvent–solvent fractionation of a crude extract, which separates a chemically diverse mixture into fractions enriched in specific classes of metabolites. Usually, each fraction presents different biological properties.^7,13–16^ Therefore, it is possible to select the fraction of interest, aiming to concentrate or isolate chemical compounds with specific bioactivity. This diversity of bioactivities reflects the distinct chemical compositions of the resulting fractions. As widely reported, polar fractions—such as those obtained with ethyl acetate and *n*-butanol—are typically enriched in flavonoids, phenolic acids, and saponins. In contrast, nonpolar fractions, including those produced with hexane, predominantly contain lipophilic constituents. Fractions of intermediate polarity, such as those obtained with dichloromethane, generally concentrate methoxylated flavonoids, sesquiterpenes, and coumarins.^17^

To determine which fraction contains the highest concentration of compounds exhibiting remarkable biological activity, a physiologically relevant model is required, and red blood cells (RBCs, or erythrocytes) meet these requirements.^18^ RBCs are enucleated cells whose primary function is gas transport,^19,20^ and hemoglobin, the protein responsible for this function, represents about 98% of RBCs’ proteins.^20^ These cells have been used as models to evaluate the cytotoxicity and biological activity of xenobiotics, such as synthetic new drugs, plant extracts, and isolated natural substances.^19,21^ Several aspects may help explain their recent scientific popularity. These cells are easy to isolate and can be obtained in large amounts since they represent about 40% of the total blood volume. Moreover, these cells do not need to be cultivated, making the assay cheaper and easier to run than experiments using cell culture.^18,22^ Despite the prevalence of hemoglobin, RBCs’ membranes include more than 340 proteins.^19^ Although RBCs are highly specialized cells, their membranes perform general functions, which makes them a representative model for studying other cell membranes.^19,21^

To evaluate the hemocompatibility or hemoprotection of xenobiotics, some biomarkers may be assessed using *in vitro* assays. Hemoglobin oxidation is particularly relevant, as reactive species generated through Fenton and Haber–Weiss chemistry readily convert oxyhemoglobin (Fe^2+^) to methemoglobin (Fe^3+^) or ferrylhemoglobin (Fe^4+^), leading to loss of its physiological oxygen-carrying function.^19,23^ Hemoglobin oxidation can also liberate its prosthetic heme group, which becomes highly reactive and unsuitable for gas transport when free in plasma,^24,25^ and may even promote the release of the iron center itself.^22,26^ Another interesting measure is the rate of lipid peroxidation, which can also lead to functional loss and cellular lysis.^22^ Nonetheless, the primary test for hemocompatibility or hemoprotection is the hemolysis assay.^19^ Hemolysis is defined as cellular injury and hemoglobin leakage, and it is one of the easiest ways to detect erythrocyte damage, since the released hemoglobin can be measured spectrophotometrically.^19,27^ Some conditions that may provoke hemolysis include mechanical and oxidative stresses, changes in acid/base balance and osmotic pressure, and xenobiotics.^19,22^

Herein, we investigated the actions of hemoprotective compounds from two complementary perspectives: their ability to modulate erythrocyte osmotic fragility (EOF) and their antioxidant properties. Antioxidants are chemical species that, even at low concentrations, can slow or inhibit chain reactions initiated by reactive species and free radicals, thereby mitigating oxidative stress.^18^ Oxidative stress arises when the production of reactive species exceeds the capacity of endogenous antioxidant defenses,^28^ a condition implicated in depression and neurodegenerative disorders,^29^ cardiovascular diseases,^30^ and cancer,^31^ and other pathologies. EOF, in turn, reflects the resistance of red blood cells (RBCs) to lysis when exposed to decreasing NaCl concentrations.^32^ As extracellular NaCl levels fall, RBCs take up water and swell, and under extreme hypotonic conditions, this leads to membrane rupture and leakage of intracellular contents.^33,34^

Nonetheless, despite previous studies on OPN, there remains a clear gap regarding the cellular antioxidant activity and hemoprotective or hemocompatibility properties of fractions derived from crude leaf extracts. Addressing this gap is essential to better understand the extent of their antioxidant effects, identify the bioactive constituents responsible, and support future technological applications of this plant matrix. In this context, this study aimed to fractionate a hydroalcoholic extract of OPN leaves to determine which fraction is richest in bioactive compounds and which exhibits the most pronounced biological activity. We hypothesized that the fractions would exhibit antioxidant and antihemolytic effects of varying intensities, enabling the identification of the most promising sample for further investigation and the potential development of a new nutraceutical.

## 2 Materials and Methods

### 2.1 Chemicals and Plant Material

Hexane and FeCl_3_.6H_2_O were purchased from Reatec (São Paulo, Brazil), while *n*-butanol, potassium persulfate, dimethyl sulfoxide (DMSO), and methanol were purchased from Synth (Diadema, Brazil). Ethyl acetate, 2,2-diphenyl-1-picrylhydrazyl (DPPH), sodium hydroxide, ethylenediaminetetraacetic acid (EDTA), disodium monohydrate acid, and methanol were purchased from Vetec (Rio de Janeiro, Brazil), and dichloromethane, hydrogen peroxide, sodium sulfate, trichloroacetic acid, NaH_2_PO_4_.H_2_O, Na_2_HPO_4_, and NaCl were obtained from Biotec (Curitiba, Brazil). Absolute ethanol, CuSO_4_.5H_2_O, and Folin–Ciocalteu reagent were purchased from Dinâmica (Indaiatuba, Brazil), while FeSO_4_.6H_2_O, ascorbic acid, and sodium carbonate were acquired from Neon (Suzano, Brazil). Gallic acid, 2-thiobarbituric acid, 2,2’-azino-bis­(3-ethylbenzothiazoline-6-sulfonic acid) (ABTS), ferrozine, pyrocatechol violet, 2,4,6-Tri­(2-pyridyl)-S-triazine (TPTZ), ascorbic acid, 2,2′-azobis­(2-amidinopropane) dihydrochloride (AAPH), and quercetin were sourced from Sigma-Aldrich (Duque de Caxias, Brazil). Sodium azide was purchased from Fmaia (Indaiatuba, Brazil). All experiments were carried out with ultrapure water (Millipore, USA).

### 2.2 Preparation of the Crude Extract and Its Fractions

The crude extract (CE) was obtained following a previously described procedure,^35^ using an optimized solvent (40% ethanol in ultrapure water) at 45 °C for 30 min. Extraction was performed by infusing 25 g of dried OPN leaves in 500 mL of solvent under magnetic stirring. After filtration, the CE was concentrated by rotary evaporation to remove the ethanol and subsequently lyophilized (−50 °C, 1200 μmHg; Terroni model LD 1500A, São Paulo, Brazil). From the resulting solid, 75.95 mg was reserved for direct CE analysis, while the remaining 2789.05 mg was dissolved in 1 L of ultrapure water.

The solubilized extract was first defatted with 250 mL of *n*-hexane and then subjected to sequential liquid–liquid partitioning with organic solvents of increasing polarity: dichloromethane, ethyl acetate, and *n*-butanol. For each solvent, 250 mL was used per cycle, and the partitioning procedure was repeated three times (Figure 1). This process yielded four fractions: dichloromethane (FD), ethyl acetate (FAE), *n*-butanol (FB), and the residual aqueous fraction (FAq), which contained the compounds remaining in the aqueous phase after *n*-butanol extraction. Organic fractions were dried over anhydrous Na_2_SO_4_, filtered, and concentrated by rotary evaporation, and FAq was freeze-dried (−50 °C, 1200 μmHg).

**Figure 1:**
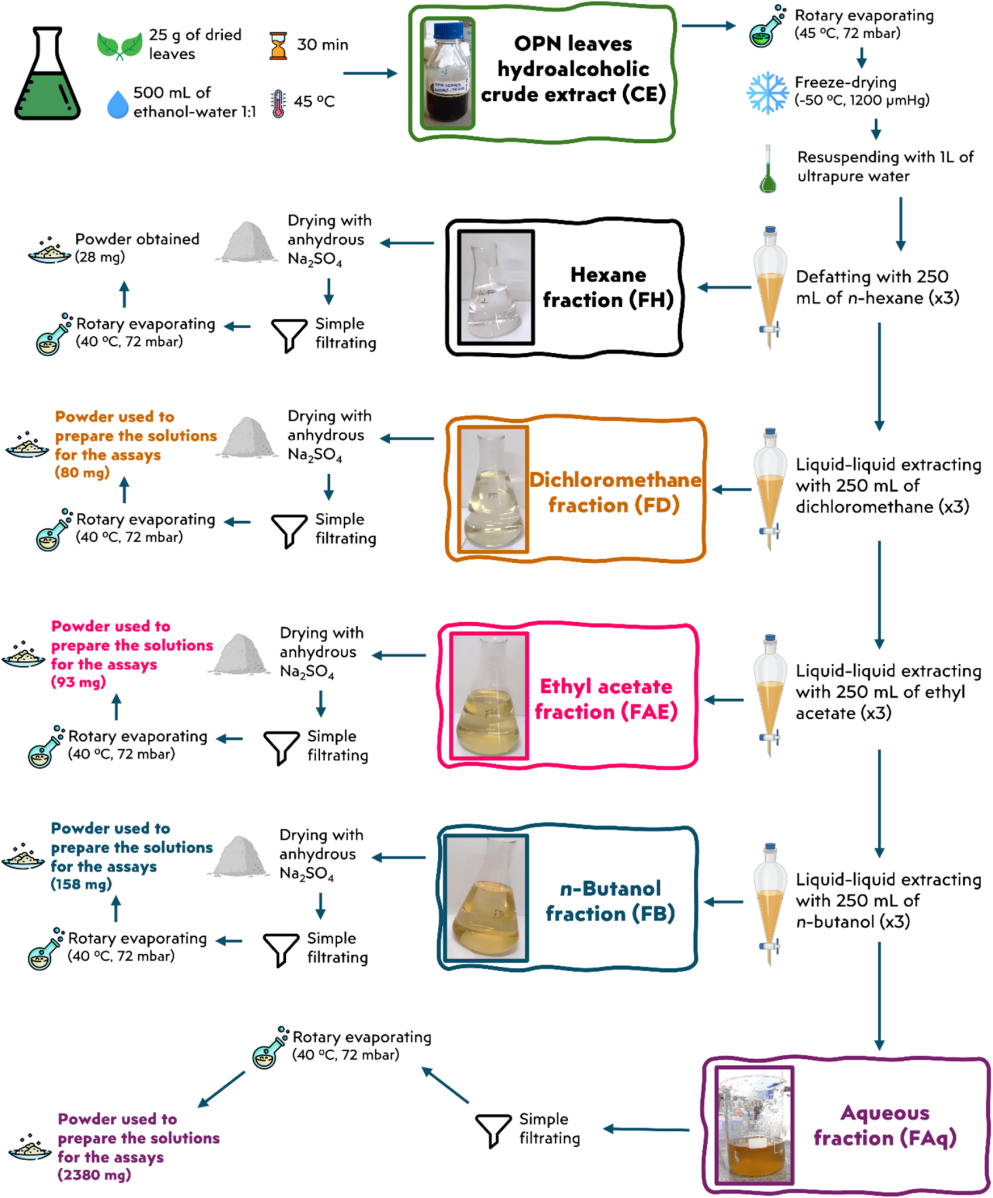
Extraction workflow representing the steps utilized to obtain the OPN leaves crude extract and its fractions.

For the chemical assays, the FD and FAE fractions were solubilized in absolute ethanol (or in HPLC-grade methanol for chromatographic analyses), whereas aqueous solutions of CE, FB, and FAq were prepared by using ultrapure water. For the biological experiments, CE, FB, and FAq were dissolved in phosphate-buffered saline (PBS, 5 mmol/L, pH 7.35, NaCl 0.9% w/v). In comparison, FD and FAE were resuspended with a 2% solution of dimethyl sulfoxide (DMSO) in PBS (5 mmol/L, pH 7.35, NaCl 0.9% w/v).

### 2.3 Chemical Composition

Total phenolic content (TPC) was determined using the Folin–Ciocalteu method,^36^ based on an analytical curve constructed with gallic acid (20–100 mg/L), and the results were expressed as mg gallic acid equivalent per g of extract or fraction (mg GAE/g). The samples’ total flavonoid content (TFC) was quantified spectrophotometrically^37^ by using an analytical curve constructed with catechin (25–300 mg/L), with results expressed as mg catechin equivalent per g of extract or fraction (mg CE/g).

High-performance liquid chromatography (HPLC) was performed on a Shimadzu LC-20AT chromatograph equipped with a diode array detector (DAD), a degasser, and an autosampler, using a reverse-phase column (C_18_, 150 × 4.6 mm^2^, particle size 3.5 μm). The extracts were filtered through a 0.22 μm nylon membrane. Chromatographic separation was performed at 40 °C, with a 10 μL injection of the samples (in triplicate) and a flow rate of 500 μL/min. The elution gradients applied were those proposed by Fidelis et al.,^38^ using a mobile phase corresponding to water acidified with 0.2% (v/v) formic acid (phase A) and acetonitrile (phase B). Rutin, chlorogenic acid, gallic acid, syringic acid, ellagic acid, ferulic acid, caffeic acid, *p*-coumaric acid, and quercetin were detected at 255, 272, 318, 325, and 360 nm, and quantified using external calibration curves (Table S1). The results were expressed as μg per gram of extract or fraction (μg/g). These compounds were selected for quantification because they have been previously reported in OPN leaves.^1,5,12,39,40^

### 2.4 Chemical Antioxidant Capacity

The 2,2-diphenyl-1-picrylhydrazyl (DPPH) radical-scavenging antioxidant capacity was measured according to the method of Brand-Williams et al.,^41^ with the results expressed as mg of ascorbic acid equivalent per g of extract or fraction (AAE/g), using an analytical curve with ascorbic acid as the standard (5–30 mg/L). The 2,2′-azino-bis­(3-ethylbenzothiazoline-6-sulfonic acid) (ABTS) cation radical-scavenging capacity was also assessed.^42^ The results were expressed as milligrams of AAE/g using an analytical curve of ascorbic acid (30–150 mg/L). Antioxidant capacity was also assessed using the ferric reducing antioxidant power (FRAP) assay,^43^ based on an ascorbic acid analytical curve (15–90 mg/L), with results likewise expressed as mg AAE/g. In addition, Fe^2+^ chelating ability was quantified according to the method of ref 44 using EDTA as the calibrator (10–50 mg/L), and results were expressed as mg of EDTA equivalents per g of extract or fraction (EDTAE/g).

### 2.5 Egg Yolk Lipoperoxidation Inhibition

Lipid protection against oxidation was assessed using the thiobarbituric acid reactive substances (TBARS) assay, which quantifies lipid peroxidation in egg yolk. Oxidative stress was induced with a 4 mmol/L FeSO_4_ solution at 37 °C for 45 min, following the method of Margraf et al.^45^ Samples were tested at concentrations (50, 100, 150, 200, and 250 μg/mL) selected based on preliminary tests and literature reports^10,46^ . The percentage of lipoperoxidation inhibition was calculated according to eq 1.

1 $$\mathrm{Inhibition}\;\left(\%\right)=\left(1-\frac{A_{\mathrm{Sample}}}{A_{\mathrm{Control}}}\right)\times 100$$

where *A*_Sample_ is the absorbance at λ = 532 nm of the samples and *A*_Control_ is the absorbance at λ = 532 nm of the control. All results were compared to the efficacy of quercetin at 10 μg/mL.

### 2.6 Protecting Red Blood Cells against Oxidative and Osmotic Injuries

#### 2.6.1 Blood Collection and Red Blood Cell Isolation

All procedures involving erythrocytes were approved by the Ethics Committee of the State University of Ponta Grossa (Certificate of Presentation for Ethical Consideration, CAAE 94830318.1.0000.0105), and free and informed written consent was obtained from the donor. O^+^ type blood samples were provided by the Wallace Thadeu de Mello e Silva Regional University Hospital. Erythrocytes were isolated by successive washes with PBS (5 mmol/L, pH 7.35, NaCl 0.9%) until a clear, colorless supernatant was obtained.^47^ In this study, repeated measurements represent technical replicates derived from the same donor sample. Our objective was to quantify assay precision, characterize erythrocyte responses under different experimental conditions, and evaluate the effects of the OPN crude extract and its fractions, rather than to infer population-level biological variability.

#### 2.6.2 H_2_O_2_-Induced Hemolysis

The protection against H_2_O_2_-induced hemolysis was assessed as described by Cruz et al.,^35^ with slight modifications. RBCs (final hematocrit 0.8%) with catalase inhibited by NaN_3_ (1 mmol/L) were oxidized with H_2_O_2_ (5 mmol/L) at 37 °C for 30, 60, 120, and 180 min, and protected by the samples at 100 μg/mL. The dose-dependent effect was also tested with samples incubated at 50, 100, and 150 μg/mL (concentrations defined by preliminary tests and based on what was found in the literature^1,10,48^ ) for only 180 min. After centrifugation (900 x*g*, 10 min), the supernatant absorbance was measured at λ = 523 nm. The negative control was obtained by replacing H_2_O_2_ and the sample with PBS; the positive control was obtained by replacing the sample with PBS; and total hemolysis (TH) was achieved by replacing the sample, NaN_3_, and H_2_O_2_ with ultrapure water. The hemolysis rate was calculated using eq 2

2 $$\mathrm{Hemolysis}\;\left(\%\right)=\left(\frac{A_{\mathrm{Sample}}}{A_{\mathrm{TH}}}\right)\times 100$$

where *A*_Sample_ is the sample’s absorbance and *A*_TH_ is the total hemolysis’ absorbance.

#### 2.6.3 Erythrocyte Osmotic Fragility (EOF)

Erythrocyte osmotic fragility (EOF) was evaluated according to the method described by de Moura et al.,^47^ with slight modifications. A hypotonic hemolysis curve was constructed by incubating red blood cells (final hematocrit 0.8%) with the samples at 100 μg/mL (or quercetin at 10 μg/mL) and phosphate buffer (5 mmol/L, pH 7.35) containing NaCl (0.2–0.9%, w/v). The absorbance was measured at λ = 523 nm, and the hemolysis rate was calculated using eq 2. TH was obtained by replacing the sample and buffer with ultrapure water. Moreover, the samples’ dose-dependent effect was evaluated at a NaCl concentration of 0.4% (w/v), with samples tested at 50, 100, and 150 μg/mL (concentrations defined by preliminary tests and based on findings in the literature^27,47^ ).

#### 2.6.4 AAPH-Induced Oxidative Stress

Oxidative stress was also induced in red blood cells (final hematocrit 5%) by incubating the samples (or quercetin) with AAPH solution (200 mmol/L) in test tubes at 37 °C in PBS. At the same time, the negative control was obtained by replacing both the AAPH solution and the samples with PBS, and the positive control was obtained by replacing only the samples with PBS. After 120 min, the tubes were centrifuged at 1200*g* for 10 min. Hemolysis was measured at λ = 523 nm, and the rates were calculated using eq 2. TH was obtained by replacing AAPH and the samples with ultrapure water. Lipoperoxidation was estimated using the thiobarbituric acid reactive substances (TBARS) method; absorbance was measured at λ = 532 nm, and inhibition was calculated according to eq 1.^22^ Hemoglobin oxidation was measured as described by Lima et al.,^49^ and the oxidation rates were calculated by using eq 3, where *A*_630nm_ is the sample’s absorbance at 630 nm and *A*_540nm_ is the sample’s absorbance at 540 nm.

3 $$\mathrm{Oxidation}\;\left(\%\right)=\left(\frac{A_{630\mathrm{nm}}}{A_{540\mathrm{nm}}}\right)\times 100$$

Advanced oxidation protein products (AOPP) were quantified according to the method described by Salam et al.,^50^ with results expressed as the percentage of AOPP generation. The calculation was performed using eq 4, where *A*_Sample_ corresponds to the absorbance of the sample at 340 nm and *A*_PositiveControl_ represents the absorbance of the positive control at the same wavelength.

4 $$\mathrm{AOPP}\;\mathrm{generation}\;\left(\%\right)=\left(\frac{A_{\mathrm{Sample}}}{A_{\mathrm{Positivecontrol}}}\right)\times 100$$

Free iron was quantified following the method described by Salam et al.,^50^ with slight modifications. Briefly, 125 μL of hemolysate was mixed with 100 μL of ascorbic acid solution (250 mg/L) in a 96-well microplate and incubated for 5 min. Then, 75 μL of ferrozine (8 mmol/L) was added, and after 30 min, the absorbance was measured at 562 nm. Results were expressed as the free iron-release rate, calculated using eq 5, where *A*_Sample_ corresponds to the sample’s absorbance and *A*_Positivecontrol_ to the absorbance of the positive control.

5 $$\mathrm{Free iron releasing}\;\left(\%\right)=\left(\frac{A_{\mathrm{Samples}}}{A_{\mathrm{Positivecontrol}}}\right)\times 100$$

### 2.7 Statistical Analysis

All assays were performed in technical triplicate, and results are presented as the mean ± standard deviation. Homoscedasticity was assessed using the Brown–Forsythe test, and differences among group means were evaluated by one-way analysis of variance (ANOVA). Pearson correlation matrices were constructed to examine the associations between the chemical composition and both chemical antioxidant and biological assay outcomes. All statistical analyses were performed using TIBCO Statistica v 13.3 software (TIBCO Software Inc., Palo Alto, USA), with a significance level of 0.05.

## 3 Results and Discussion

### 3.1 Phenolic Composition

The TPC values are shown in Table 1 and range from 34 to 65 mg of GAE/g. FAE and FB were the fractions richest in polyphenols, with FAE exhibiting a greater TPC than CE. In the literature, the phenolic content of hydroalcoholic extracts of OPN leaves ranges from 24 to 64 mg GAE/g, depending on the ethanol proportion used in the extraction solvent,^10,35^ which is consistent with the values obtained for CE in the present study. Regarding the fractions, previous work reported TPC values of 49, 4, and 2 mg tannic acid equivalents (TAE)/g for the dichloromethane, ethyl acetate, and hydromethanolic fractions, respectively.^13^

**Table 1:** Total Phenolic (TPC) and Flavonoid (TFC) Contents and Individual Phenolic Composition Assessed by HPLC/DAD/UV of the OPN Leaves Hydroalcoholic Crude Extract (CE) and Its Dichloromethane (FD), Ethyl Acetate (FAE), *n*-Butanol (FB), and Aqueous (FAq) Fractions^a^,^b^

Sample	FD	FAE	FB	FAq	CE	*p*-Value homoscedasticity	*p*-Value ANOVA
Yield (mg)	80	93	158	2380	2865	--	--
Yield (%)	<1	<1	1	9	11	--	--
TPC (mg GAE/g)	34 ± 1^c^	65 ± 2^a^	58 ± 3^ab^	38 ± 2^c^	54 ± 3^b^	0.809	≤0.050
TFC (mg CE/g)	26 ± 1^c^	56 ± 2^a^	28 ± 1^c^	27 ± < 1^c^	31 ± 1^b^	0.528	≤0.050
Gallic acid (μg/g)	ND	<LOQ	763 ± 3^b^	880 ± 10^a^	808 ± 74^ab^	0.472	≤0.050
Chlorogenic acid (μg/g)	ND	5147 ± 266^a^	ND	ND	52 ± 5^b^	0.155	≤0.050 (paired *t*-test)
Syringic acid (μg/g)	314 ± 21	ND	ND	ND	<LOD	NA	NA
Caffeic acid (μg/g)	ND	ND	329 ± 4	ND	<LOD	NA	NA
*p*-Coumaric acid (μg/g)	258 ± 25^e^	10625 ± 234^a^	1144 ± 1^b^	381 ± 21^d^	508 ± 26^c^	0.061	≤0.050
Ferulic acid (μg/g)	1001 ± 10^b^	18482 ± 680^a^	739 ± 10^d^	614 ± 40^e^	854 ± 18^c^	0.434	≤0.050
Ellagic acid (μg/g)	82 ± < 1^d^	36402 ± 302^a^	5524 ± 130^c^	ND	8792 ± 80^b^	0.234	≤0.050
Quercetin (μg/g)	577 ± 8^b^	1491 ± 127^a^	ND	ND	<LOD	0.545	≤0.050 (paired *t*-test)
Rutin (μg/g)	41 ± 4^e^	1691 ± 21^d^	3889 ± 0.1^a^	2510 ± 27^c^	3368 ± 82^b^	0.530	≤0.050

a Different letters in the same row represent statistically different results (*p* ≤ 0.05).

b GAE = gallic acid equivalents; CE = catechin equivalents.

The TFC of fractions ranged from 26 to 56 mg of CE/g (Table 1), and FAE exhibited the highest content. Previously, several fractions of the methanolic extract of OPN leaves had their flavonoid content quantified, and values reported ranged between 8.33 and 54.58 mg rutin equivalent per g.^13^ Due to the differing standards used in the calibration curve, it was not possible to determine whether the contents found in this work exceeded those reported in the literature.

Previous studies have reported that OPN leaf extracts contain predominantly hydroxycinnamic acids, flavonoids, and hydroxybenzoic acids.^1,5,12,39,40^ Herein, the individual phenolic composition of each fraction varied according to its polarity (Table 1). Gallic acid, for instance, was detected in all samples except for FD, and its content increased with the polarity of the fraction. This trend is expected, given the compound’s structural features, a small aromatic ring substituted with three hydroxyl groups, which confer high polarity and, consequently, greater solubility in aqueous media. Earlier reports indicated gallic acid levels of 4.7–6.2 μg/g in OPN leaves,^12^ whereas in our study, its content ranged from 763 to 880 μg/g in the extracts or fractions.

Rutin was detected in all samples, with concentrations ranging from 41 μg/g (FD) to 3889 μg/g (FB), in agreement with the findings of Jacobsen et al.^39^ Notably, rutin was also predominant in the FAq. Reported values in the literature range from 0.5–5.3 mg/g in fractions and 6.6–10.78 mg/g in crude extracts.^35,39^ On the other hand, caffeic acid was detected exclusively in FB, which is consistent with its intermediate polarity as a hydroxycinnamic acid, allowing efficient interaction with *n-*butanol. Its content (329 μg/g) closely matched the ∼300 μg/g previously reported for this fraction^39^ and exceeded the previously described for crude hydroalcoholic extracts of OPN leaves (40–126 μg/g).^51^

Ferulic (614–18482 μg/g) and *p*-coumaric acids (258–10625 μg/g) were also detected in all samples, and the contents of both were greater in FAE than in the other samples, which agrees with previous reports.^39^ Chlorogenic acid, in turn, was detected only in this fraction and in CE (52–5147 μg/g), whereas earlier studies reported contents ranging from 0.88 to 4.22 mg/100 g in OPN leaves.^5^ These compounds are hydroxycinnamic acids, which possess a longer hydrocarbon chain than hydroxybenzoic acids, e.g., gallic acid. This structural difference increases their hydrophobicity and helps explain the variation in their distribution across fractions of differing polarity.

Ellagic acid was detected in all samples except FAq, with contents ranging from 82 to 36 402 μg/g, substantially higher than previously reported values for OPN leaves,^1^ and exceeding the 899–1702 μg/g described for *Pereskia grandifolia* leaf extracts.^52^ Quercetin, the only aglycone evaluated in this study, was found exclusively in FD and FAE, the least polar fractions, with concentrations of 577 and 1491 μg/g, respectively, both markedly higher than the 40–100 μg/g previously reported.^39^ Syringic acid, a methoxylated hydroxybenzoic acid, was identified only in FD, consistent with its low polarity, and occurred at 314 μg/g, surpassing the 33 μg/g reported for a 75% hydroalcoholic extract of OPN leaves.^53^

### 3.2 Chemical Antioxidant Capacity

Regarding the DPPH scavenging capacity (Table 2), CE exhibited an activity of 41 mg AAE/g, a value similar to that reported during extraction optimization (44 mg AAE/g).^35^ Across all samples, free radical-scavenging capacity ranged from 14 to 79 mg of AAE/g, with FAE showing the highest efficiency and FD the lowest. Comparable trends have been reported for other plant matrices. For example, in a hydroalcoholic extract of lemongrass leaves (*Cymbopogon citratus* DC.), Hacke et al.^54^ observed that the chloroform fraction presented the lowest activity (IC_50_ > 100 μg/mL), followed by the aqueous fraction (IC_50_ = 51 μg/mL), with the ethyl acetate showing the highest activity (IC_50_ = 34 μg/mL). Similarly, for a methanolic extract of perilla leaves (*Perilla frutescens* L.), while the ethyl acetate (IC_50_ ∼ 30 μg/mL) and *n*-butanol (IC_50_ ∼ 70 μg/mL) fractions showed antioxidant activity, the dichloromethane and aqueous fractions were ineffective (IC_50_ > 150 μg/mL).^55^ Collectively, these findings align with the pattern observed in the present study, in which the ethyl acetate fraction demonstrated the highest antioxidant capacity, followed by *n*-butanol, aqueous, and finally dichloromethane.

**Table 2:** Chemical Antioxidant Capacity of the OPN Leaves Hydroalcoholic Crude Extract (CE) and Its Dichloromethane (FD), Ethyl Acetate (FAE), *n*-Butanol (FB), and Aqueous (FAq) Fractions^a^,^b^

Sample	DPPH (mg AAE/g)	ABTS (mg AAE/g)	FRAP (mg AAE/g)	Fe^2+^ chelating (mg EDTAE/g)
FD	14 ± 1^d^	30 ± 2^c^	31 ± 1^c^	36 ± 3^c^
FAE	79 ± 2^a^	114 ± 5^a^	152 ± 7^a^	12 ± 1^e^
FB	41 ± 1^b^	55 ± 4^b^	58 ± 4^b^	17 ± 1^d^
FAq	25 ± 1^c^	34 ± 1^c^	28 ± 1^c^	67 ± 2^b^
CE	41 ± 1^b^	64 ± 4^b^	50 ± 1^b^	96 ± 1^a^
*p*-Value homoscedasticity	0.896	0.633	0.427	0.765
*p*-Value ANOVA	≤0.050	≤0.050	≤0.050	≤0.050

a Different letters in the same column represent statistically different results (*p* ≤ 0.05).

b DPPH = 2,2-diphenyl-1-picrylhydrazyl; ABTS = 2,2′-azino-bis­(3-ethylbenzothiazoline-6-sulfonic acid); FRAP = ferric reducing antioxidant potential; AAE = ascorbic acid equivalents; and EDTAE = EDTA equivalents.

For ABTS^•+^ scavenging (Table 2), CE exhibited an activity of 64 mg of AAE/g. Across the samples, the results ranged from 30 to 114 mg AAE/g, with FD and FAq showing the lowest efficiencies and FAE presenting the highest. Similar trends have been reported for other plant matrices. In the ethanolic extract of *Gynura procumbens* Merr. leaves, for example, the ethyl acetate fraction displayed the strongest activity (IC_50_ = 0.2 mg/mL), followed by *n*-butanol (IC_50_ = 0.7 mg/mL) and chloroform (IC_50_ = 3.3 mg/mL),^56^ mirroring the polarity-dependent pattern observed in the present study. Regarding FRAP values (Table 2), CE exhibited 50 mg AAE/g, slightly lower than the 62 mg AAE/g previously shown by this extract.^35^ Consistent with the other antioxidant assays, FAE presented the highest reducing power (152 mg of AAE/g), whereas FD and FAq showed the lowest values (31 and 28 mg of AAE/g, respectively).

CE presented a Fe^2+^ chelation efficiency of 96 mg EDTAE/g (Table 2), a value notably higher than the 31 mg EDTAE/g previously reported for the hydroalcoholic extract of OPN leaves.^35^ Among the fractions, chelation capacity ranged from 12 to 67 mg of EDTAE/g, with FAq being the most efficient and FAE the least effective. Comparable behavior has been described for *P. frutescens* L. leaf extracts, in which the dichloromethane, ethyl acetate, and aqueous fractions displayed chelation efficiencies of approximately 20% at 200 μg/mL, while the *n*-butanol fraction reached around 40%.^55^

These results indicated that FAE had the greatest reducing potential, but FAq was the most efficient at chelating transition metals. This divergence is expected, as the assays probe distinct antioxidant mechanisms and the performance of each sample is closely linked to its chemical composition and structural features. In particular, the degree of substitution on the aromatic ring and the position of functional groups can markedly influence both reducing power and metal-chelating capacity, enhancing or diminishing these activities depending on the molecular architecture.^57^

The DPPH and ABTS scavenging capacity and FRAP values were positively correlated with the samples’ total phenolic and flavonoid contents, as well as their levels of ellagic acid, quercetin, chlorogenic acid, *p*-coumaric acid, and ferulic acid. In contrast, metal-chelating ability was associated with the gallic acid content (Table S2). Although the apparent association with these phenolic compounds shown by the Pearson correlation, additional experiments are required to confirm causality, as antioxidant properties in complex plant matrices may arise from multiple compounds and can be influenced by additive, antagonistic, or synergistic interactions among polyphenols.^58,59^ Nevertheless, if confirmed, these correlations would not be surprising, given that phenolic compounds are recognized antioxidant substances and may act by several antioxidant mechanisms.^11,60,61^

### 3.3 Lipid Oxidation Inhibition

In the egg yolk model (Figure 2), samples at 100 μg/mL exhibited inhibition efficiencies above 40%, but only FD (55%) and FAE (53%) were as effective as quercetin 10 μg/mL (56%) (Table 3). Interestingly, FD and CE did not display dose-dependent behavior, likely due to the complex chemical composition of these samples: depending on concentration, the antioxidant effects of certain polyphenols may be counterbalanced by antagonistic interactions with other secondary metabolites, leading to a nonmonotonic response. FAE showed the highest overall efficiency among the samples, surpassing quercetin at 10 μg/mL only at the highest tested concentration (250 μg/mL). FB matched the activity of quercetin at 10 μg/mL only when concentrated to 200 and 250 μg/mL (47% and 56%, respectively). On the other hand, FAq showed inhibition levels statistically comparable to quercetin at 150, 200, and 250 μg/mL (49%, 56%, and 60%, respectively).

**Figure 2:**
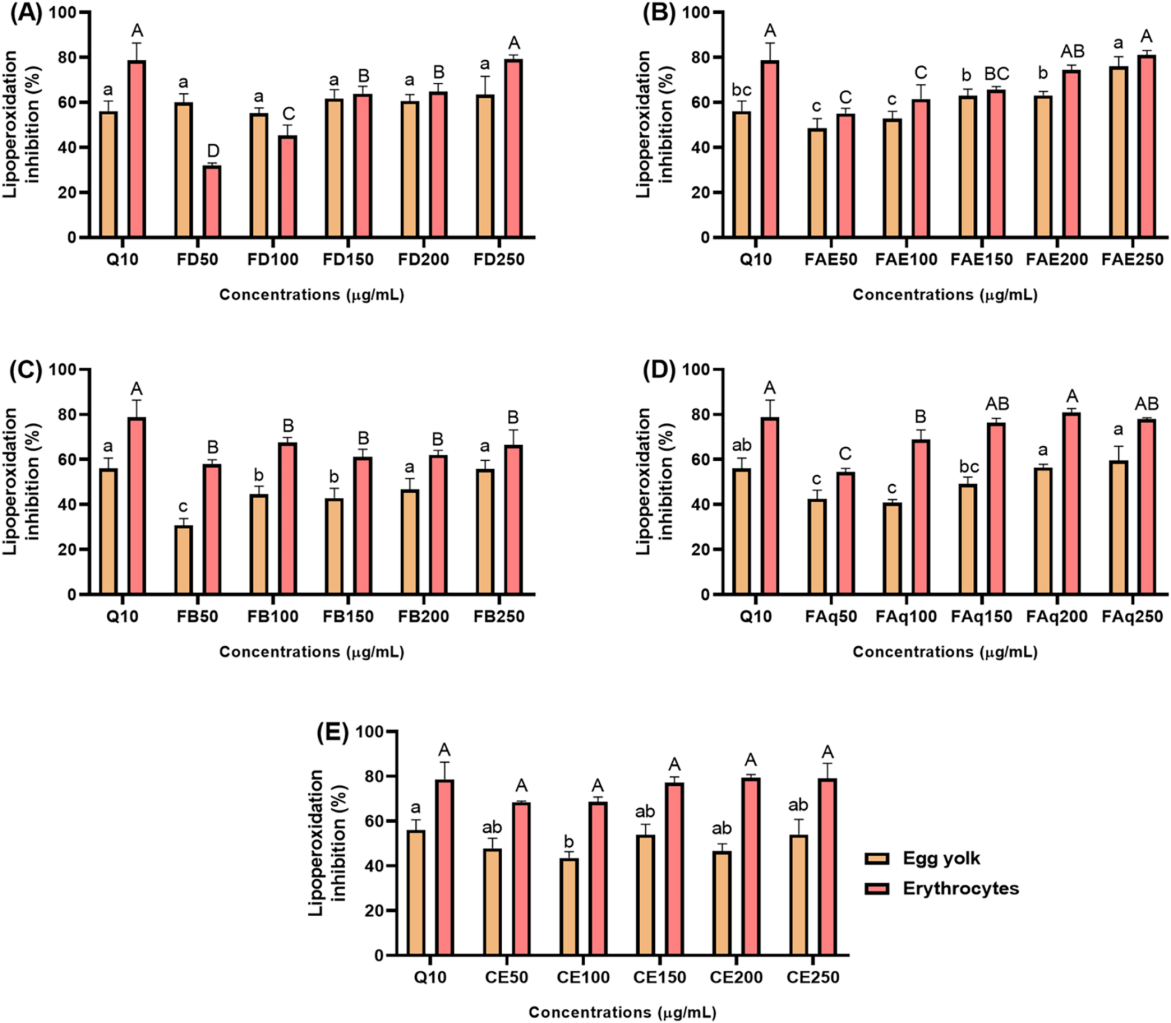
Lipoperoxidation inhibition in egg yolk and erythrocytes by the OPN leaves crude extract (CE, 2E) and its dichloromethane (FD, 2A), ethyl acetate (FAE, 2B), *n*-butanol (FB, 2C), and aqueous (FAq, 2D) fractions. Different capital letters indicate statistically different lipoperoxidation inhibition values (*p* ≤ 0.05) in erythrocytes. Different lowercase letters refer to statistically different lipoperoxidation inhibition rates in egg yolk (*p* ≤ 0.05).

**Table 3:** Protective Effect of the OPN Leaves Hydroalcoholic Crude Extract (CE) and Its Dichloromethane (FD), Ethyl Acetate (FAE), *n*-Butanol (FB), and Aqueous (FAq) Fractions (All Samples Concentrated at 100 μg/mL) on Egg Yolk and RBC against Oxidative and Osmotic Stresses^a^

	Lipoperoxidation inhibition		Oxidative hemolysis (120 min)		Protein oxidation markers in RBC			EOF	
Sample (100 μg/mL)	Egg yolk (%)	RBC (%)	H_2_O_2_-induced (%)	AAPH-induced (%)	AOPP (%)	Hemoglobin oxidation (%)	Free iron (%)	H_50_ (%)	Hemolysis (%, [NaCl] = 0.4%)
FD	54.8 ± 2.6^a^	45.3 ± 4.6^c^	25.5 ± 2.2^b^	2.8 ± 0.5^e^	78.6 ± 6.8^b^	46.1 ± 1.3^a^	65.1 ± 0.9^bc^	0.406 ± 0.004^d^	50.3 ± 3.1^d^
FAE	52.8 ± 3.2^a^	61.5 ± 6.4^b^	14.5 ± 0.6^e^	4.8 ± 0.4^de^	79.7 ± 2.6^b^	34.8 ± 2.3^bc^	50.7 ± 3.0^e^	0.401 ± 0.011^d^	45.2 ± 4.8^d^
FB	44.6 ± 3.5^b^	67.5 ± 2.3^b^	16.5 ± 1.9^de^	7.5 ± 1.5^d^	72.3 ± 2.3^b^	39.8 ± 2.8^b^	60.2 ± 6.2^cd^	0.424 ± 0.003^bc^	61.8 ± 7.2^c^
FAq	40.8 ± 1.4^b^	68.8 ± 4.3^ab^	22.2 ± 1.5^bc^	20.3 ± 4.0^b^	79.2 ± 3.8^b^	30.7 ± 3.0^cd^	70.8 ± 5.2^b^	0.450 ± 0.013^a^	74.3 ± 1.7^b^
CE	43.5 ± 2.8^b^	68.6 ± 2.2^ab^	19.7 ± 0.4^cd^	11.0 ± 2.6^c^	55.7 ± 2.7^c^	27.7 ± 0.6^d^	54.7 ± 4.4^de^	0.447 ± 0.006^a^	75.2 ± 0.9^ab^
Quercetin 10 μg/mL	56.1 ± 4.5^a^	78.7 ± 7.6^a^	17.0 ± 0.8^de^	2.3 ± 0.1^ef^	70.4 ± 6.0^b^	38.0 ± 3.9^b^	37.8 ± 1.1^f^	0.406 ± 0.010^cd^	50.9 ± 4.0^d^
Positive control	--	--	31.6 ± 0.3^a^	48.4 ± 0.6^a^	100.0 ± 4.8^a^	49.1 ± 1.2^a^	100.0 ± 6.8^a^	0.435 ± 0.009^ab^	84.3 ± 2.8^a^
Negative control	--	--	2.2 ± 0.2^f^	0.9 ± 0.1^f^	43.0 ± 3.6^d^	5.3 ± 1.6^e^	27.0 ± 2.4^g^	--	--
*p*-Value homoscedasticity	0.143	0.188	0.462	0.132	0.757	0.803	0.065	0.629	0.332
*p*-Value ANOVA	≤0.05	≤0.05	≤0.05	≤0.05	≤0.05	≤0.05	≤0.05	≤0.05	≤0.05

a RBC = red blood cells or erythrocytes; AAPH = 2,2′-azobis­(2-methylpropionamidine) dihydrochloride; EOF = erythrocyte osmotic fragility; AOPP = advanced oxidation protein products. Different letters in the same column represent statistically different results (*p* ≤ 0.05).

The lower efficiency of the fractions and the crude extract compared to quercetin can be attributed to the fact that our samples are complex matrices of secondary metabolites. Some of which may act as antagonists to the antioxidant compounds, while others may be inert, meaning that higher concentrations of plant material are required to match or surpass the efficiency of an isolated antioxidant.^59^

In RBCs, at 100 μg/mL (Table 3), only FD and FAE exhibited an efficiency comparable to quercetin at 10 μg/mL (79%). Both FB and CE provided dose-independent protection, although CE remained less effective than quercetin at all tested concentrations. In contrast, FD, FAE, and FAq presented dose-dependent protection. FD at 250 μg/mL (79%) matched the efficiency of quercetin, while FAE and FAq achieved comparable protection at 200 and 150 μg/mL, respectively.

Overall, these results show that FD and FAE were the most effective samples for protecting lipids from oxidation in egg yolk, whereas FB, FAq, and CE were the most efficient at inhibiting lipoperoxidation in RBCs. The lipoperoxidation inhibition capacity of the samples was correlated with their rutin (*r*_RBCs_ = 0.822), syringic acid (*r*_eggyolk_ = 0.618), quercetin (*r*_eggyolk_ = 0.822), and gallic acid (*r*_RBCs_ = 0.764) contents. These compounds possess well-established antioxidant properties and have been previously reported as effective inhibitors of lipid peroxidation;^22^ however, additional experiments are required to confirm whether they are, indeed, the primary contributors to the protective effects.

Formerly, in egg yolk, OPN leaf extracts obtained using water, ethanol, acetone, and binary or ternary mixtures of these solvents were reported to inhibit lipoperoxidation by 14–36%.^35^ Similarly, for hydroalcoholic extract fractions (70% ethanol) of lemongrass (*C. citratus*) leaves, the most efficient sample was that obtained with ethyl acetate (∼20 nmol MDA/g), followed by chloroform fractions (∼30 nmol MDA/g) and aqueous (∼50 nmol MDA/g).^54^ In RBCs, the hydroalcoholic extract of OPN leaves was previously shown to reduce TBARS formation by approximately 30–40%,^1^ whereas the hydroalcoholic extract of jabuticaba (*Myrciaria cauliflora* vari. Sabará) leaves diminished the lipoperoxidation by about 20%.^46^

### 3.4 Oxidative Hemolysis

Plant-sourced antioxidants may protect RBCs against H_2_O_2_– and AAPH-induced hemolysis through several mechanisms: (1) scavenging ROS or preventing their generation, (2) interacting with membrane proteins, particularly band 3, to occupy structural gaps generated during oxidative stress, or (3) decreasing the membrane’s fluidity by forming hydrogen bonds with the polar heads of phospholipids.^22^ Overall, the samples were more effective against AAPH-induced damage than H_2_O_2_-induced hemolysis (Figure 3), as reflected by the more pronounced reduction in hemolysis rates under the AAPH challenge. FAE again stood out as having the greatest efficacy in protecting RBCs. This difference arises from the mechanism underlying hemolysis: H_2_O_2_ primarily oxidizes hemoglobin and other intracellular proteins,^21,62^ whereas AAPH generates peroxyl radicals that primarily attack membrane lipids.^22,63^ Thus, our results suggest that the OPN samples inhibit oxidative hemolysis more efficiently through suppression of lipid peroxidation than through protection against protein oxidation.

**Figure 3:**
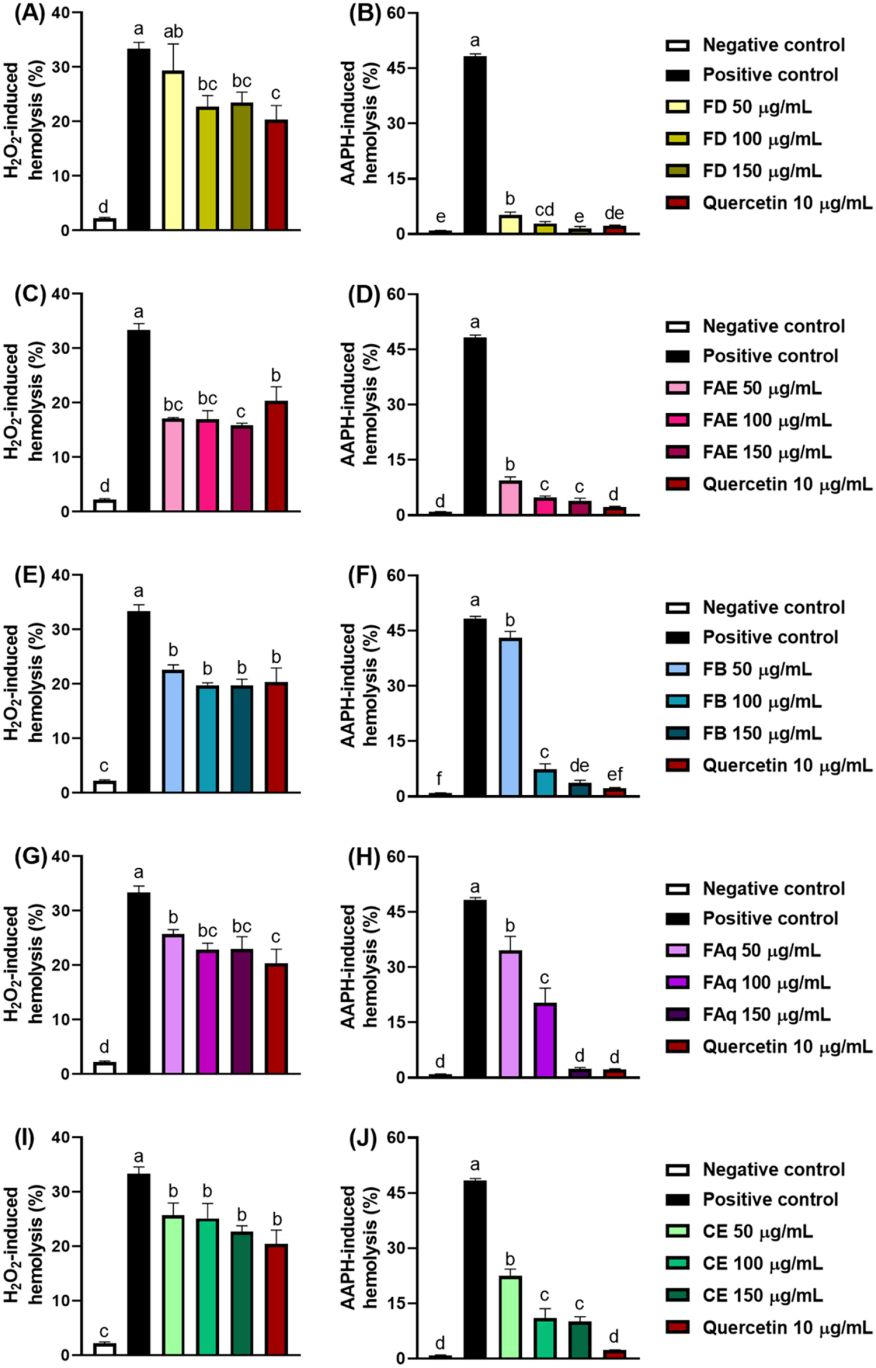
Dose-dependent protection against H_2_O_2_– and AAPH-induced oxidative hemolysis by the OPN leaves hydroalcoholic crude extract (I and J) and its dichloromethane (A and B), ethyl acetate (C and D), *n*-butanol (E and F), and aqueous (G and H) fractions. Different letters reveal statistically different averages (*p* ≤ 0.05).

#### 3.4.1 H_2_O_2_-Induced Hemolysis

H_2_O_2_ is an inexpensive oxidant naturally generated in the process of cellular respiration. Through Fenton-type reactions, it can generate hydroxyl radicals, which readily oxidize components of the erythrocyte membrane.^21^ In the present study, H_2_O_2_-induced hemolysis correlated with the samples’ total phenolic, total flavonoid, ellagic acid, quercetin, chlorogenic acid, *p*-coumaric acid, and ferulic acid contents (Table S2). This pattern suggests that higher concentrations of these compounds may contribute to lower hemolysis rates when RBCs are incubated with the samples. However, validation assays are still required to confirm these trends.

CE was effective in protecting erythrocytes at all of the tested reaction times (Figure 4E). This protection was dose-independent and comparable to that of quercetin at 30 and 180 min. Among the fractions, FD provided significant protection at 30, 120, and 180 min; however, at 60 min, its hemolysis rate did not differ from the control (19%). This pattern likely reflects the early dynamics of the reaction: although protection is present at the beginning, its magnitude may be too small to produce a statistically distinguishable effect. As the reaction progresses, the antioxidant activity becomes more pronounced, allowing the samples’ protective effects to emerge more clearly.

**Figure 4:**
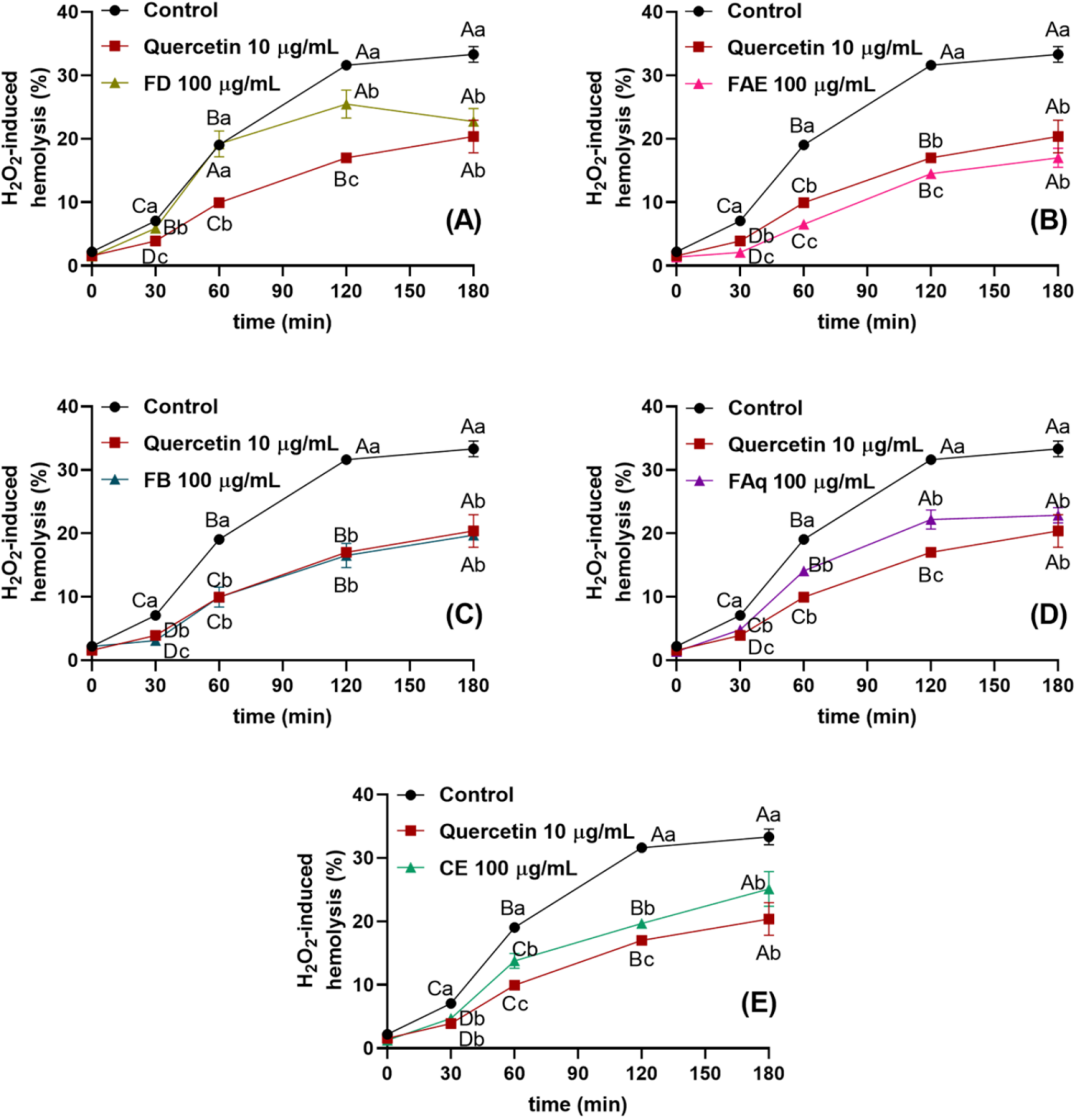
H_2_O_2_-induced oxidation kinetics in the presence of dichloromethane (A), ethyl acetate (B), *n*-butanol (C), and aqueous (D) fractions of the OPN leaves crude extract (E). Different capital letters indicate statistically different hemolysis values (*p* ≤ 0.05) for each sample at different reaction times. Different lowercase letters indicate hemolysis rates in the presence and absence of an antioxidant, at each reaction time, statistically different results (*p* ≤ 0.05).

On the other hand, FAE protected RBCs from oxidative stress at all exposure times, exhibiting an efficiency superior to that of quercetin 10 μg/mL, except at 180 min. FB also protected RBCs at all tested times (except 30 min) and concentrations, with an intensity comparable to that of the standard. FAq inhibited hemolysis at all tested incubation times and concentrations, acting in a dose-independent manner. For comparison, our research group previously reported that OPN leaf extracts inhibited only 5–20% of H_2_O_2_-induced hemolysis.^35^ However, information regarding the antihemolytic activity of the OPN fractions remains scarce.

#### 3.4.2 AAPH-Induced Hemolysis

2,2′-azobis­(2-amidinopropane) dihydrochloride, or just AAPH, is a synthetic azo compound that generates peroxyl radicals upon thermal decomposition, inducing oxidative stress that targets membrane lipids and ultimately leads to hemolysis.^22^ All of the samples protected RBCs against hemolysis at every tested concentration, displaying a dose-dependent effect (Figure 3). FD was the most effective sample, showing protection comparable to quercetin at 100 and 150 μg/mL, and even achieving a hemolysis rate similar to the negative control at 150 μg/mL. FAE also demonstrated strong activity, reducing hemolysis from approximately 50% (positive control) to about 10% at the lowest tested concentration (50 μg/mL).

At 50 μg/mL, FB was the least efficient sample. However, at 100 and 150 μg/mL, the protection provided by this sample was stronger, and at the highest tested concentration (150 μg/mL), the FB efficacy was similar to that of quercetin (10 μg/mL). FAq also exerted dose-dependent protection; in fact, at 150 μg/mL, the hemolysis rate did not differ from that observed with quercetin or the negative control, reinforcing the antihemolytic potential of this fraction. Regarding CE, no difference in efficacy was observed between 100 and 150 μg/mL; nonetheless, at both concentrations, CE was more effective than that at 50 μg/mL.

Previously, the protection of RBCs against AAPH-induced hemolysis by OPN leaf extracts was evaluated by Garcia et al.,^10^ who reported IC_50_ values ranging from 57 to 131 μg/mL, depending on the incubation time. In the present study, hemolysis inhibition correlated negatively with the samples’ quercetin content (*r* = −0.564), suggesting that higher quercetin levels may contribute to greater antihemolytic protection. This association is plausible, given that quercetin is a well-established antihemolytic compound.^22,48^

### 3.5 Protein Oxidation Markers in Erythrocytes

#### 3.5.1 Advanced Oxidation Protein Products (AOPP)

Under oxidative stress, RBC proteins are susceptible to oxidation, generating several chromophoric byproducts that absorb at 340 nm, such as pentosidine, carbonyls, and proteins cross-linked by dityrosine, and these components are denominated advanced oxidation protein products (AOPP).^64^ All fractions protected RBCs against protein oxidation with similar intensity, whereas CE showed the greatest efficiency (Table 3). FAq was the only sample that did not exhibit dose-dependent protection. FD and FAE, at 50 and 100 μg/mL (Figure 5), reduced AOPP generation to levels comparable to quercetin, and at 150 μg/mL, both fractions showed even greater protection than the standard. FB also provided protection equivalent to quercetin at all tested concentrations, with its highest efficiency observed at 150 μg/mL. The AOPP generation was correlated with the samples’ rutin content (*r* = −0.560), indicating that higher rutin content was associated with lower AOPP generation. This finding is consistent with previous reports demonstrating that rutin can inhibit AOPP formation in RBCs under oxidative stress.^50^

**Figure 5:**
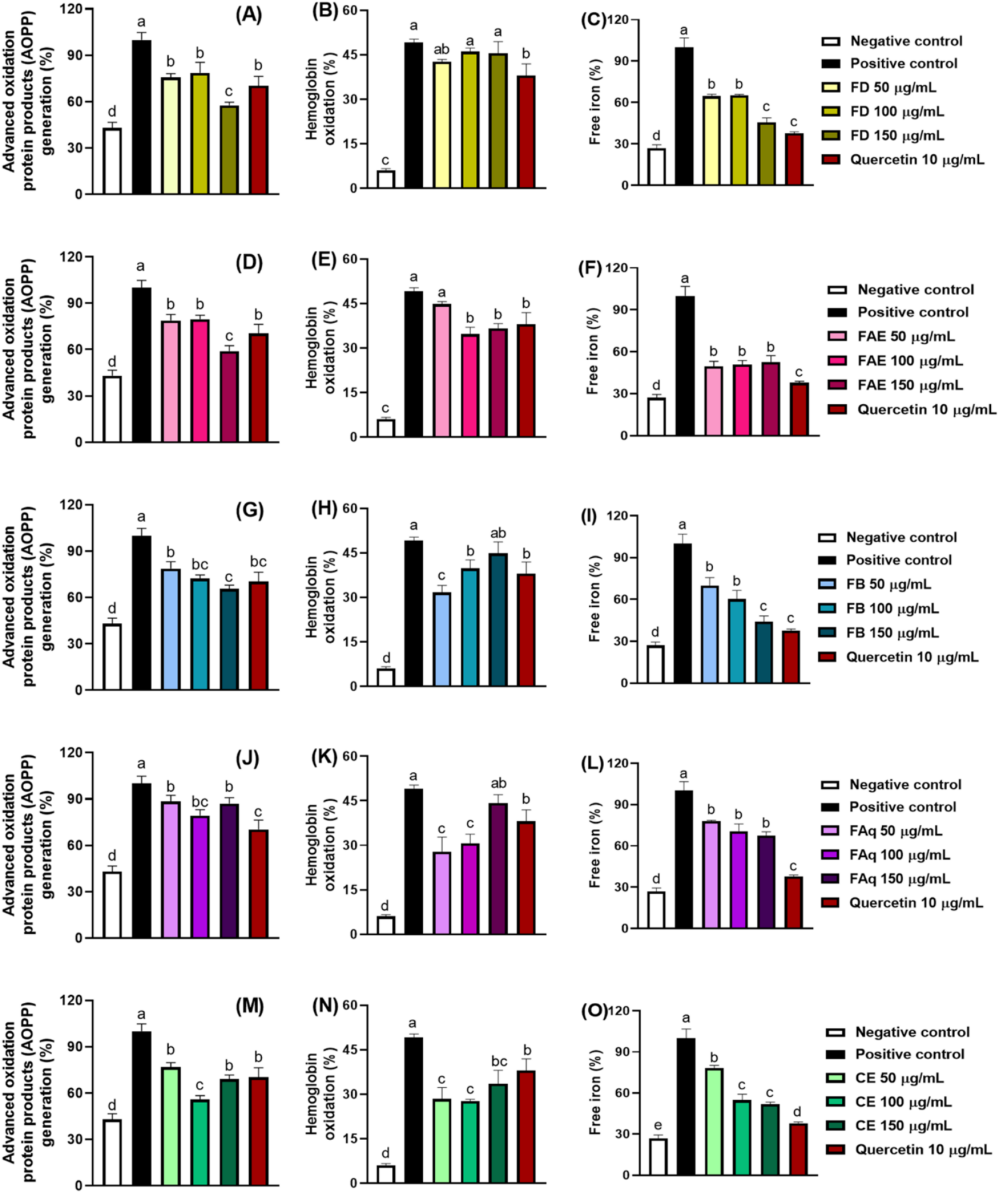
Erythrocyte protein oxidation markers in the presence and absence (positive control) of the OPN leaves hydroalcoholic crude extract (M, N, and O) and its dichloromethane (A, B, and C), ethyl acetate (D, E, and F), *n*-butanol (G, H, and I), and aqueous (J, K, and L) fractions. Different letters indicate statistically different averages (*p* ≤ 0.05).

#### 3.5.2 Hemoglobin Oxidation

Hemoglobin oxidation is a key consequence of oxidative stress in RBCs.^49^ When oxyhemoglobin (Fe^2+^) is oxidized to methemoglobin (Fe^3+^), the molecule loses its ability to transport oxygen; therefore, protecting hemoglobin from oxidation is essential for maintaining erythrocyte function.^23^ The efficiency of fractions in preventing hemoglobin oxidation is shown in Figure 5. CE protected RBCs at every tested concentration, whereas FD was the only sample that failed to provide protection, as its hemoglobin oxidation rates did not differ from those of the positive control at any concentration. FAE did not exhibit protective activity at the lowest tested concentration (50 μg/mL), but at 100 and 150 μg/mL, its hemoglobin oxidation rates were statistically indistinguishable from those observed with quercetin at 10 μg/mL. At 150 μg/mL, FB and FAq were ineffective at protecting hemoglobin from oxidation. However, at lower concentrations (50 and 100 μg/mL), the hemoglobin oxidation rates were lower than those in the positive control. It occurs because, up to a certain concentration, the antioxidant effects of polyphenols may prevail, while as the concentration increases, the antagonistic effects of other secondary metabolites may become more pronounced.^59^ Our results indicated that hemoglobin oxidation rates were correlated with the rutin and gallic acid contents of the samples (Table S2). However, as with the other correlations observed in this study, additional experiments are necessary to independently confirm this association. For comparison, an ethanolic extract of *Moringa oleifera* Lam. leaves was previously shown to protect hemoglobin from oxidation, reducing methemoglobin levels from 1.510% to 0.163–0.643% in a dose-dependent manner.^65^

#### 3.5.3 Free Iron

The release of free iron in RBCs is a consequence of iron-binding protein oxidation, such as hemoglobin, ferritin, and transferrin.^26^ Free iron can further exacerbate oxidative stress by participating in Fenton reactions,^19^ and, to reduce free iron rates, antioxidants may inhibit protein oxidation and/or chelate the iron released from these proteins.^22,26^ Among the samples, FAE and CE were the most effective, as RBCs incubated with these fractions exhibited the lowest iron-release rates (Table 3). FD, FB, and CE displayed dose-dependent behavior, whereas the protection provided by FAE and FAq was not concentration-dependent (Figure 5). Free-iron levels were negatively correlated with the samples’ total phenolic, ellagic acid, chlorogenic acid, *p*-coumaric acid, and ferulic acid contents (Table S2), indicating that higher concentrations of these compounds were associated with reduced iron release under AAPH-induced oxidative conditions.^50^

#### 3.5.4 Erythrocyte Osmotic Fragility (EOF)

Hypotonic hemolysis curves were recorded for all samples (100 μg/mL) by varying the NaCl concentration from 0.2% to 0.9% (Figure 6). These curves usually exhibit sigmoidal behavior, and the graphs can be used to evaluate the hemoprotective capacity of the samples using several methods. Herein, we utilized two parameters: H_50_ and hemolysis rate at [NaCl] = 0.4%. These choices were made because the 0.4% NaCl concentration is the stage of hypotonic hemolysis where the samples’ dose dependency is usually most evident, and H_50_ is the NaCl concentration when the hemolysis rate reaches 50%.^34,47^

**Figure 6:**
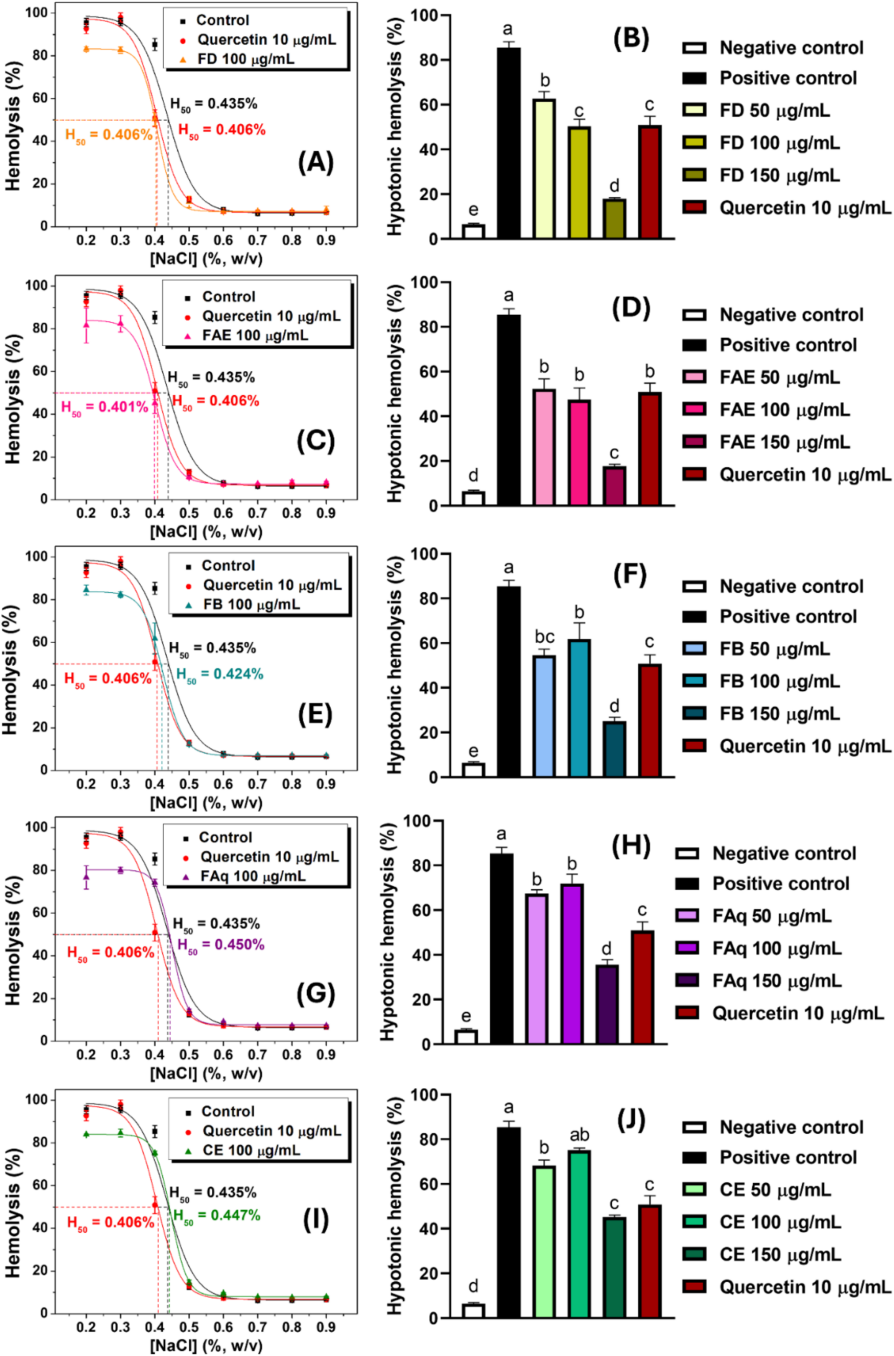
Curves of hypotonic hemolysis and dose-dependent effect at [NaCl] = 0.4% (w/v) of OPN leaves hydroalcoholic crude extract (I and J) and its dichloromethane (A and B), ethyl acetate (C and D), *n*-butanol (E and F), and aqueous (G and H) fractions. Different letters reveal statistically different responses (*p* ≤ 0.05).

FD and FAE were the most effective samples, reducing both the H_50_ value and the hemolysis rate with the same efficiency as quercetin (Table 3). In the meantime, while FAq lowered the hemolysis rate but not the H_50_, CE was unable to mitigate both parameters. All samples presented dose-dependent protection (Figure 6), and notably, the fractions were more effective at 150 μg/mL than quercetin. Although OPN leaf extracts have previously been reported to possess antihemolytic activity, particularly hydroalcoholic extracts,^10,35^ this is the first study to demonstrate such activity in fractions derived from a crude extract. H_50_ values were correlated with their quercetin content, while the hypotonic hemolysis responses were associated with the ellagic acid, quercetin, chlorogenic acid, *p*-coumaric acid, and ferulic acid contents of the samples (Table S2). Several of these phenolic compounds have previously been reported as antihemolytic agents,^34,48,66^ acting by stabilizing the cell membrane through hydrogen bonds with membrane phospholipids or by occupying structural defects generated by osmotic stress.^33,35^

## 4 Conclusion

The CE fractions were rich in phenolics and flavonoids and exhibited antioxidant and antihemolytic activities, protecting RBCs against oxidative and hypotonic stresses. Nonetheless, FAE emerged as one of the most effective fractions across all of the assays performed in this study. This fraction contained the highest levels of several key bioactive compounds, including chlorogenic acid, *p*-coumaric acid, quercetin, ferulic acid, and ellagic acid, and effectively protected RBCs against H_2_O_2_- and AAPH-induced oxidative stress. In addition to reducing lipid and protein oxidation, FAE also mitigated EOF by enhancing the membrane stability.

This study represents an initial data-gathering effort aimed at understanding the biological potential of a crude extract and its fractions. Although the results are highly promising, they remain preliminary and do not guarantee the safety or efficacy of these samples. Additional investigations using more complex biological models, assessments of bioaccessibility and bioavailability of the bioactive compounds, and in vivo studies are still required. Based on the evidence presented here, FAE appears to be the fraction with the most potent biological activity and is a promising candidate for the development of a nutraceutical or food additive. Nonetheless, the remaining fractions should not be disregarded, as they also demonstrated significant antioxidant activity and may likewise be suitable for incorporation into new products if their bioactivity is further validated.

## Supplementary Material



## Notes

Additional information regarding the analytical curves, including the work range, LOD, and LOQ. Chromatograms and Pearson’s correlation coefficients (PDF)
