# Supplementary material for: Solvent–Solvent Fractionation of Ora-Pro-Nobis (Pereskia aculeata) Leaves Enhances Polyphenol Enrichment and Red Blood Cell Protection against Oxidative and Osmotic Stress
Source: ACS Omega. 2026 Mar 19;11(12):19292–306. doi: 10.1021/acsomega.5c12684 (PMC13044836; doi:10.1021/acsomega.5c12684)

Solvent-solvent fractionation of ora-pro-nobis (*Pereskia aculeata*) leaves enhances polyphenol enrichment and red blood cell protection against oxidative and osmotic stress

Thiago M. Cruz [a]*, Yasmin Stelle [a], Daniel Granato [b], Mariza B. Marques [a]

[a] Department of Chemistry, State University of Ponta Grossa, Av. Carlos Cavalcanti, 4748, 84030-900, Ponta Grossa, Brazil. *E-mail: mcruz.thiago01@gmail.com

[b] School of Science, Auckland University of Technology, Auckland 1010, New Zealand.

Table S1 – Retention time (t_R), wavelength used to detect, analytical curve, and limits of detection (LOD) and quantification (LOQ) for each phenolic compound investigated in ora-pro-nobis leaves by HPLC-DAD-UV.

Peak	t_R (min)	Compound	Detection λ (nm)	Analytical curve	R^2	LOD (mg/L)	LOQ (mg/L)
1	7.72	Gallic acid	272	y = 56600x + 24052	0.998	0.018	0.055
2	17.08	Rutin	360	y = 35743x – 1028	0.999	0.036	0.111
3	17.52	Ellagic acid	360	y = 42138x – 8717.5	0.999	0.023	0.072
4	19.52	Quercetin	360	y = 76546x – 7062	0.999	0.010	0.031
5	26.10	Chlorogenic acid	325	y = 63781x – 7231.5	0.999	0.014	0.042
6	26.66	Caffeic acid	325	y = 105042x – 53002	0.997	0.006	0.018
7	29.68	*p*-Coumaric acid	318	y = 124348x + 142935	0.995	0.219	0.503
8	30.15	Ferulic acid	325	y = 105233x – 17124	0.999	0.041	0.125

Table S2 – Pearson's correlation matrices between the phenolic composition and the chemical antioxidant and hemoprotector activities.

	DPPH	ABTS	FRAP	Fe^{2+} chelating	Egg yolk TBARS	RBC TBARS	AAPH-induced haemolysis	H_2O_2-induced haemolysis	Haemoglobin oxidation	AOPP	Free iron	H_{50}	Hypotonic hemolysis
**TPC**	*r* = 0.896	*r* = 0.859	*r* = 0.779	*r* = -0.343	*r* = 0.035	*r* = 0.349	*r* = -0.285	*r* = -0.588	*r* = -0.284	*r* = -0.279	*r* = -0.757	*r* = -0.391	*r* = -0.119
	p = <0.001	*p* = <0.001	*p* = 0.001	*p* = 0.210	*p* = 0.901	*p* = 0.203	*p* = 0.304	*p* = 0.021	*p* = 0.305	*p* = 0.314	*p* = 0.001	*p* = 0.150	*p* = 0.476
**TFC**	*r* = 0.926	*r* = 0.947	*r* = 0.972	*r* = -0.446	*r* = 0.398	*r* = 0.018	*r* = -0.327	*r* = -0.636	*r* = -0.157	*r* = 0.166	*r* = -0.664	*r* = -0.448	*r* = -0.563
	p = <0.001	*p* = <0.001	*p* = <0.001	*p* = 0.096	*p* = 0.141	*p* = 0.950	*p* = 0.234	*p* = 0.011	*p* = 0.576	*p* = 0.555	*p* = 0.007	*p* = 0.094	*p* = 0.029
**Rutin**	*r* = 0.338	*r* = 0.216	*r* = 0.034	*r* = 0.234	*r* = -0.780	*r* = 0.822	*r* = 0.424	*r* = 0.017	*r* = -0.611	*r* = -0.560	*r* = -0.313	*r* = 0.441	*r* = 0.638
	p = 0.258	*p* = 0.476	*p* = 0.911	*p* = 0.442	*p* = 0.002	*p* = 0.001	*p* = 0.149	*p* = 0.957	*p* = 0.027	*p* = 0.046	*p* = 0.298	*p* = 0.131	*p* = 0.019
**Ellagic acid**	*r* = 0.961	*r* = 0.978	*r* = 0.988	*r* = -0.445	*r* = 0.372	*r* = 0.043	*r* = -0.374	*r* = -0.657	*r* = -0.167	*r* = 0.093	*r* = -0.742	*r* = -0.502	*r* = -0.546
	p = <0.001	*p* = <0.001	*p* = <0.001	*p* = 0.097	*p* = 0.172	*p* = 0.878	*p* = 0.169	*p* = 0.008	*p* = 0.552	*p* = 0.742	*p* = 0.002	*p* = 0.057	*p* = 0.035
**Quercetin**	*r* = 0.732	*r* = 0.785	*r* = 0.873	*r* = -0.606	*r* = 0.734	*r* = -0.430	*r* = -0.564	*r* = -0.651	*r* = 0.236	*r* = 0.429	*r* = -0.522	*r* = -0.655	*r* = -0.830
	p = 0.003	*p* = 0.001	*p* = <0.001	*p* = 0.022	*p* = 0.003	*p* = 0.125	*p* = 0.036	*p* = 0.012	*p* = 0.416	*p* = 0.126	*p* = 0.055	*p* = 0.011	*p* = <0.001
**Gallic acid**	*r* = -0.269	*r* = -0.363	*r* = -0.509	*r* = 0.591	*r* = -0.890	*r* = 0.764	*r* = 0.765	*r* = 0.428	*r* = -0.617	*r* = -0.450	*r* = 0.299	*r* = 0.784	*r* = 0.902
	p = 0.332	*p* = 0.183	*p* = 0.053	*p* = 0.020	*p* = <0.001	*p* = 0.001	*p* = 0.001	*p* = 0.112	*p* = 0.014	*p* = 0.092	*p* = 0.279	*p* = 0.001	*p* = <0.001
**Chlorogenic acid**	*r* = 0.886	*r* = 0.907	*r* = 0.968	*r* = -0.532	*r* = 0.453	*r* = -0.051	*r* = -0.356	*r* = -0.704	*r* = -0.061	*r* = 0.301	*r* = -0.603	*r* = -0.466	*r* = -0.636
	p = <0.001	*p* = <0.001	*p* = <0.001	*p* = 0.041	*p* = 0.090	*p* = 0.857	*p* = 0.193	*p* = 0.003	*p* = 0.830	*p* = 0.275	*p* = 0.017	*p* = 0.080	*p* = 0.011
**Caffeic acid**	*r* = 0.021	*r* = -0.074	*r* = -0.061	*r* = -0.437	*r* = -0.227	*r* = 0.234	*r* = -0.153	*r* = -0.279	*r* = 0.249	*r* = -0.043	*r* = -0.005	*r* = -0.056	*r* = 0.018
	p = 0.940	*p* = 0.795	*p* = 0.828	*p* = 0.103	*p* = 0.417	*p* = 0.402	*p* = 0.586	*p* = 0.313	*p* = 0.371	*p* = 0.880	*p* = 0.985	*p* = 0.842	*p* = 0.950
***p*-Coumaric acid**	*r* = 0.909	*r* = -0.922	*r* = -0.979	*r* = -0.570	*r* = 0.436	*r* = -0.037	*r* = -0.366	*r* = -0.736	*r* = -0.055	*r* = 0.292	*r* = -0.623	*r* = -0.477	*r* = -0.638
	p = <0.001	*p* = <0.001	*p* = <0.001	*p* = 0.026	*p* = 0.104	*p* = 0.895	*p* = 0.180	*p* = 0.002	*p* = 0.844	*p* = 0.292	*p* = 0.013	*p* = 0.072	*p* = 0.010
**Ferulic acid**	*r* = 0.882	*r* = 0.905	*r* = 0.964	*r* = -0.541	*r* = 0.469	*r* = -0.084	*r* = -0.370	*r* = -0.705	*r* = -0.047	*r* = 0.301	*r* = -0.611	*r* = -0.486	*r* = -0.649
	p = <0.001	*p* = <0.001	*p* = <0.001	*p* = 0.037	*p* = 0.078	*p* = 0.766	*p* = 0.175	*p* = 0.003	*p* = 0.867	*p* = 0.275	*p* = 0.015	*p* = 0.066	*p* = 0.009

Figure S1 – Chromatograms of phenolic compounds detected at (A) 272 nm, (B) 360 nm, (C) 318 nm, and (D) 325 nm in OPN leaves extracts and fractions. The numbering of peaks is described in Table S1.

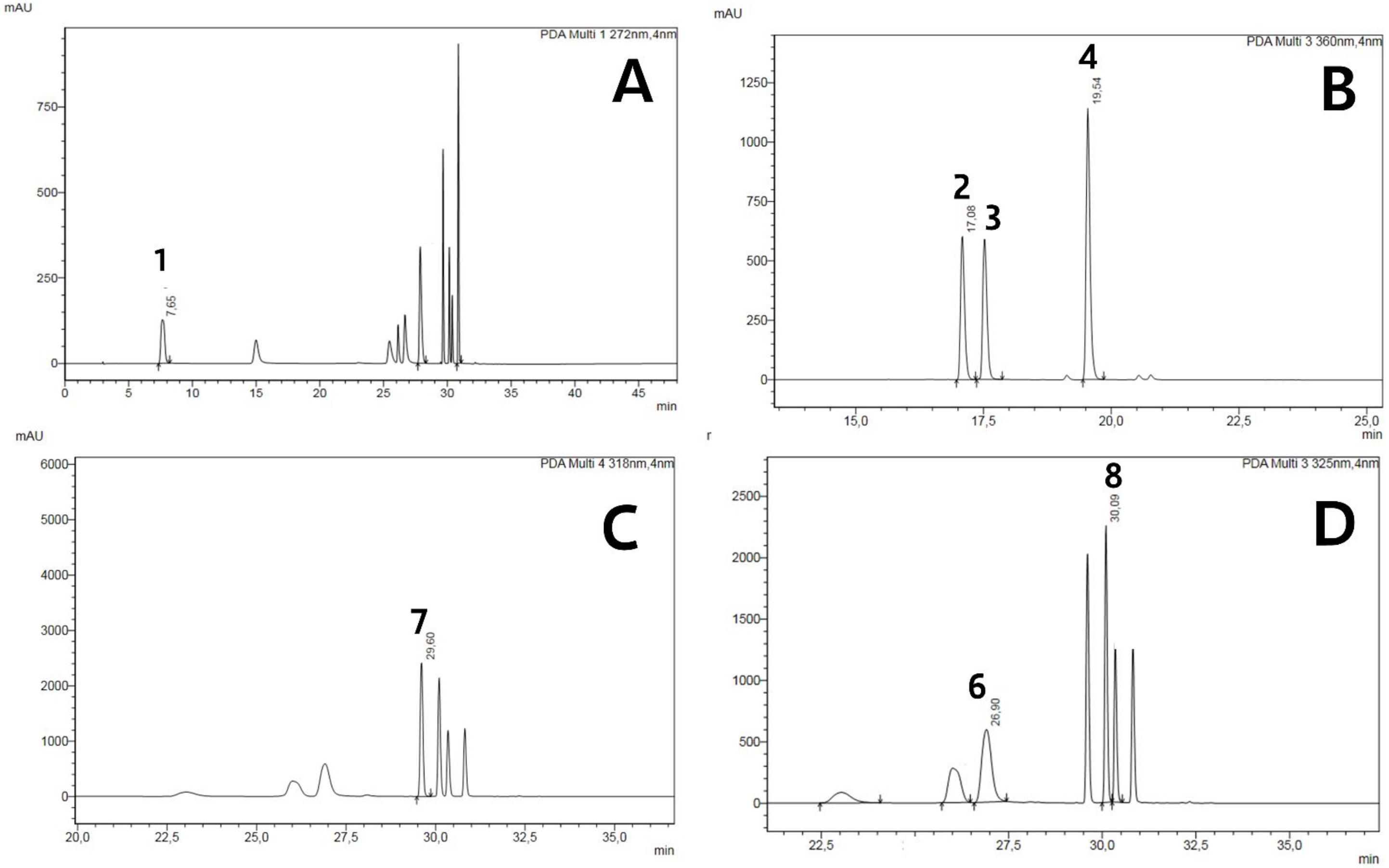

Supplement: Supplementary file 1 [file ao5c12684_si_001.pdf]
